# Supplementary material for: Ciliary and rhabdomeric photoreceptor-cell circuits form a spectral depth gauge in marine zooplankton
Source: eLife. 2018 May 29;7:e36440. doi: 10.7554/eLife.36440 (PMC6019069; doi:10.7554/eLife.36440)
Supplement: Transparent reporting form [file elife-36440-transrepform.docx]

***eLife’s* transparent reporting form**

We encourage authors to provide detailed information *within their submission* to facilitate the interpretation and replication of experiments. Authors can upload supporting documentation to indicate the use of appropriate reporting guidelines for health-related research (see [EQUATOR Network](http://www.equator-network.org/%20)), life science research (see the [BioSharing Information Resource](https://biosharing.org/)), or the [ARRIVE guidelines](http://www.plosbiology.org/article/info:doi/10.1371/journal.pbio.1000412) for reporting work involving animal research. Where applicable, authors should refer to any relevant reporting standards documents in this form.

If you have any questions, please consult our Journal Policies and/or contact us: [editorial@elifesciences.org](mailto:editorial@elifesciences.org).

**Sample-size estimation**

- You should state whether an appropriate sample size was computed when the study was being designed
- You should state the statistical method of sample size computation and any required assumptions
- If no explicit power analysis was used, you should describe how you decided what sample (replicate) size (number) to use

Please outline where this information can be found within the submission (e.g., sections or figure legends), or explain why this information doesn’t apply to your submission:

Experiments with batches of larvae (Figure 3, 4) were designed to include data from <7 batches coming from separate fertilization events, each preferably from different days.

Calcium imaging experiments were done on individually mounted larvae. Multiple mRNA injection experiments were done and the indicated number of larvae were tested on different days (Figure 2). Experiments were designed to produce internally consistent data to allow the assessment of general patterns. Wild type and c-opsin1 mutant larvae were first imaged for calcium signals (Figure 4) and then subjected to genotyping individually.

Connectome data: due to the significant time and resources needed to generate these data we only analyzed one specimen.

**Replicates**

- You should report how often each experiment was performed
- You should include a definition of biological versus technical replication
- The data obtained should be provided and sufficient information should be provided to indicate the number of independent biological and/or technical replicates
- If you encountered any outliers, you should describe how these were handled
- Criteria for exclusion/inclusion of data should be clearly stated
- High-throughput sequence data should be uploaded before submission, with a private link for reviewers provided (these are available from both GEO and ArrayExpress)

Please outline where this information can be found within the submission (e.g., sections or figure legends), or explain why this information doesn’t apply to your submission:

During calcium imaging experiments larvae that showed low general GCaMP6 fluorescence due to insufficient amounts of mRNA injected or low expression were excluded from the measurements. Larvae that showed developmental defects or severe movement artefacts were also excluded. Biological replicates correspond to individual larvae.

During behavioral experiments, each batch contained approximately 50-200 larvae. For statistical analyses, results from single larvae from the same batch were pooled and were considered as one biological replicate.

The number of biological replicates is indicated in the figure legends.

During the behavioural experiments, we excluded batches that had a low number of larvae due to problems with fertilisation or development. We only tested healthy batches where most of the larvae showed robust phototactic behaviour, assessed qualitatively by directional white light illumination.

**Statistical reporting**

- Statistical analysis methods should be described and justified
- Raw data should be presented in figures whenever informative to do so (typically when N per group is less than 10)
- For each experiment, you should identify the statistical tests used, exact values of N, definitions of center, methods of multiple test correction, and dispersion and precision measures (e.g., mean, median, SD, SEM, confidence intervals; and, for the major substantive results, a measure of effect size (e.g., Pearson's r, Cohen's d)
- Report exact p-values wherever possible alongside the summary statistics and 95% confidence intervals. These should be reported for all key questions and not only when the p-value is less than 0.05.

Please outline where this information can be found within the submission (e.g., sections or figure legends), or explain why this information doesn’t apply to your submission:

The statistical tests are described in the figure legends. For all graphs we provided the source data files.

(For large datasets, or papers with a very large number of statistical tests, you may upload a single table file with tests, Ns, etc., with reference to sections in the manuscript.)

**Group allocation**

- Indicate how samples were allocated into experimental groups (in the case of clinical studies, please specify allocation to treatment method); if randomization was used, please also state if restricted randomization was applied
- Indicate if masking was used during group allocation, data collection and/or data analysis

Please outline where this information can be found within the submission (e.g., sections or figure legends), or explain why this information doesn’t apply to your submission:

Batches were allocated to mutant and wildtype groups based (Figure 4). Batches were grouped based on developmental stage in Figure 3. During spectral measurements, wavelengths were not randomized, but dark periods were introduced between different wavelengths. During calcium imaging, larvae were analyzed individually.

**Additional data files (“source data”)**

- We encourage you to upload relevant additional data files, such as numerical data that are represented as a graph in a figure, or as a summary table
- Where provided, these should be in the most useful format, and they can be uploaded as “Source data” files linked to a main figure or table
- Include model definition files including the full list of parameters used
- Include code used for data analysis (e.g., R, MatLab)
- Avoid stating that data files are “available upon request”

Please indicate the figures or tables for which source data files have been provided:

Source data are provided for all the graphs and calcium imaging traces.
